# Pericardioesophageal fistulae after left atrial ablation: a case series

**DOI:** 10.1093/jscr/rjae160

**Published:** 2024-03-17

**Authors:** Savan K Shah, Audrey C Pendleton, Arsalan A Khan, Gillian C Alex

**Affiliations:** Department of Cardiovascular and Thoracic Surgery, Rush University Medical Center, Chicago, IL 60605, USA; Department of Cardiovascular and Thoracic Surgery, Rush University Medical Center, Chicago, IL 60605, USA; Department of Cardiovascular and Thoracic Surgery, Rush University Medical Center, Chicago, IL 60605, USA; Department of Cardiovascular and Thoracic Surgery, Rush University Medical Center, Chicago, IL 60605, USA

**Keywords:** pericardioesophageal fistula, left atrial ablation, iatrogenic, esophagus, left atrium, cardiothoracic, surgery

## Abstract

Pericardioesophageal fistula is an uncommon, yet serious complication that can occur after left atrial ablation for cardiac arrhythmias. Timing of this complication is variable; however, it has been reported to occur from a week to over a month post-ablation. The incidence of this complication after ablation is <0.05%; however with increasing rates of left atrial ablations, early recognition is imperative. Nonspecific symptoms, including chest pain, dysphagia, and fever, can indicate the presence of a fistula within the first month after ablation. Early drainage with subsequent definitive treatment is key to limiting morbidity. Here we report four cases of pericardioesophageal fistula all occurring ~1 month post-ablation, with two patients surviving after prompt diagnosis and surgical treatment. Successful treatment in these two cases was achieved with fistula takedown and intercostal muscle flap interposition and esophageal stenting.

## Introduction

Left atrial ablation for atrial fibrillation is a common and generally well-tolerated procedure with incidence of thermal injury to the esophagus occurring in <0.05% of cases [1–4]. However, such an injury can result in devastating complications including fistulization of the esophagus to the heart or pericardium, which carries mortality up to 80% [5, 6]. Given the typically delayed onset of symptoms and variable presentation, a high index of suspicion is required in order to enact timely intervention and decrease the risk of death. Here we present four cases of pericardioesophageal fistulae, their courses, interventions, and outcomes.

## Case series

### Case one

**Figure 1:**
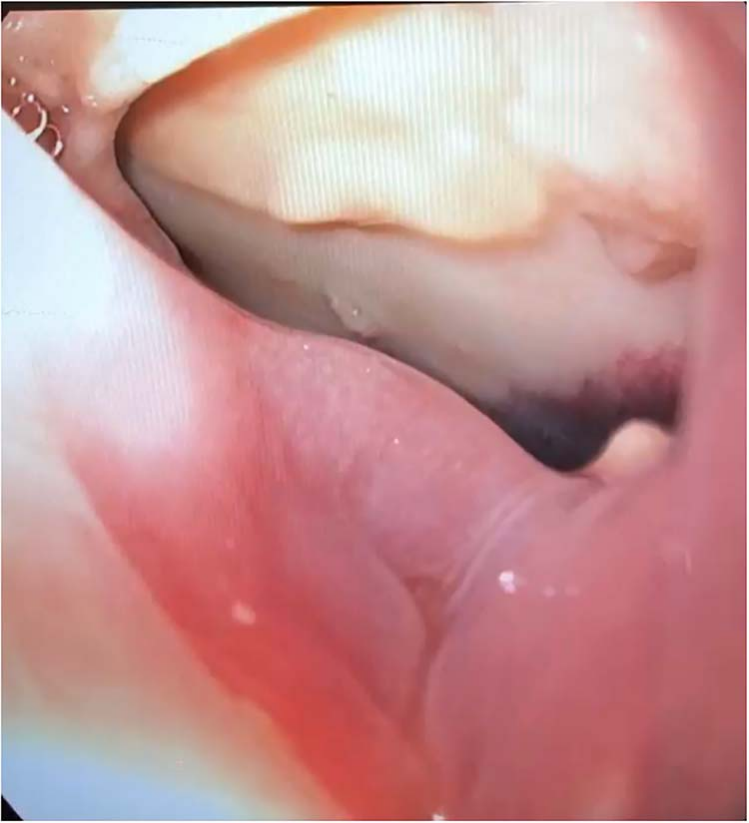
Endoscopic view of pericardioesophageal fistula of Case 1.

A 63-year-old man developed chest pain and fevers 3 days after left atrial ablation for paroxysmal atrial fibrillation and was readmitted. Computed tomography (CT) angiography at that time showed loculated fluid along the right lateral margin of the distal esophagus and a small pericardial effusion. He was treated for presumed pneumonia and discharged after 3 days. Over the following 2 weeks, he developed progressive dysphagia and persistent chest pain. Subsequent evaluation with a CT scan of the chest demonstrated pneumopericardium and a large pericardial effusion concerning for fistula formation. He was transferred to our institution 11 days after initial presentation for definitive management. Upon arrival to the cardiac intensive care unit (ICU), the patient was awake and alert, normotensive, but tachycardic. He was evaluated by both cardiac and thoracic surgery and taken emergently to the operating room (OR) for an exploratory thoracotomy, at which point he went into cardiac arrest and an emergent sternotomy was performed. Upon entering the chest, 1 L of purulent pericardial fluid and succus was drained, and the patient was placed on cardiopulmonary bypass (CPB). Examination of the heart demonstrated no injury; however, a fistula was identified posteriorly in the pericardium that communicated with the esophagus. An upper endoscopy was performed, and a 3 cm anterior perforation was visualized (Fig. 1). Given the instability of the patient, repair was deferred, and his mediastinum was widely drained. The patient could not be weaned from CPB due to hemodynamic instability and he was placed on veno-arterial extracorporeal membrane oxygenation with his chest left open and packed. Overnight, the patient developed fixed and dilated pupils. A non-contrast CT of the brain demonstrated infratentorial herniation and the patient was transitioned to comfort care and later expired.

### Case two

A 64-year-old man developed pleuritic chest pain 4 weeks after his left atrial ablation for atrial fibrillation and was found to have pericardial fluid and gas on a CT scan of the chest. He underwent a pericardiocentesis 21 h after initial presentation for cardiogenic shock in the setting of a pericardial effusion. He underwent a subxiphoid pericardial window 2 days later with drainage of purulent fluid, as well as endoscopic suturing of a 6 mm esophageal perforation identified incidentally at 35 cm from the incisors during percutaneous endoscopic gastrostomy tube placement. A left chest tube was placed for a large effusion and broad-spectrum antibiotics were started. A subsequent esophagram demonstrated a small, but persistent leak through a fistula between his esophagus and pericardium. He was subsequently transferred to our institution 10 days after initial presentation. Prior to transfer, he became altered and pulled out his pericardial drain and gastrostomy tube.

On arrival, he was discovered to be septic with an elevated white blood cell count and tachycardia with continued altered mental status. He was taken emergently to the OR for a left thoracotomy and upper endoscopy, which revealed a small hole in his stomach as well as healthy mucosa at the site of previous suture repair with no obvious perforation. A left posterolateral thoracotomy was then performed, and fibrous adhesions were taken down. A large pericardial window was performed and loculated purulent material was removed from the pericardial sac. The esophagus was inspected and dissected free from the pericardium and found to be viable. Drains were left in the pericardium and left chest, and an esophageal stent was placed over the sutured perforation endoscopically. The patient then underwent an exploratory laparotomy with repair of the stomach and placement of a gastrojejunostomy tube.

The pericardial and pleural cultures grew polymicrobial bacteria and fungi, so the patient continued antibiotics and antifungals for 6 weeks. His postoperative course was significant for a recurrent left pleural effusion, which was drained, and atrial fibrillation, which was controlled with anti-arrhythmic medication. An esophagram performed on postoperative Day (POD) 6 was negative for any esophageal or gastric leak. He was discharged on POD 14 and returned for an outpatient upper endoscopy and stent removal 1 month after stent placement.

### Case three

A 70-year-old man, with a history of atrial fibrillation and left atrial ablation, presented 4 weeks post-ablation with dyspnea and chest pain. He underwent a CT angiography of the chest which demonstrated a moderate-sized pericardial effusion and a small amount of air. Initial transthoracic echocardiogram showed no evidence of cardiac tamponade. He experienced acute clinical decompensation from early cardiac tamponade discovered on transesophageal echocardiogram 72 h after initial presentation, with subsequent pericardiocentesis and drain placement. The purulent fluid evacuated grew *Klebsiella* and *Candida* and treatment with broad-spectrum antibiotics and antifungals was started. An esophagram was significant for a pericardioesophageal fistula and he was transferred to our institution for definitive management 8 days after initial presentation.

Upon arrival, the patient was hemodynamically stable, but with acute renal failure not requiring dialysis. The patient was taken to the OR emergently, where he underwent a right posterolateral thoracotomy. The pericardium was entered, and purulent fluid was drained. A 1 cm defect in the lateral wall of the upper esophagus was discovered after the esophagus was dissected free from the pericardium and was extended to expose the mucosa completely. After debriding devitalized tissue, the esophageal injury was repaired primarily in two layers and reinforced with the intercostal muscle flap. Drains were placed, and the patient returned to the ICU in stable condition. The patient initially did well, but later had worsening tachycardia and persistent leukocytosis. His esophagram was negative for a leak; however a CT scan of the chest revealed a moderate pericardial effusion. He was taken back to the OR for a left anterior mini-thoracotomy and pericardial window. Purulent fluid was again encountered and evacuated from the pericardium. Drains were placed, and the patient was extubated in the OR. The remainder of his course was significant for persistent atrial fibrillation, for which he underwent cardioversion. He was discharged to a nursing home within 4 weeks of admission.

### Case four

**Figure 2:**
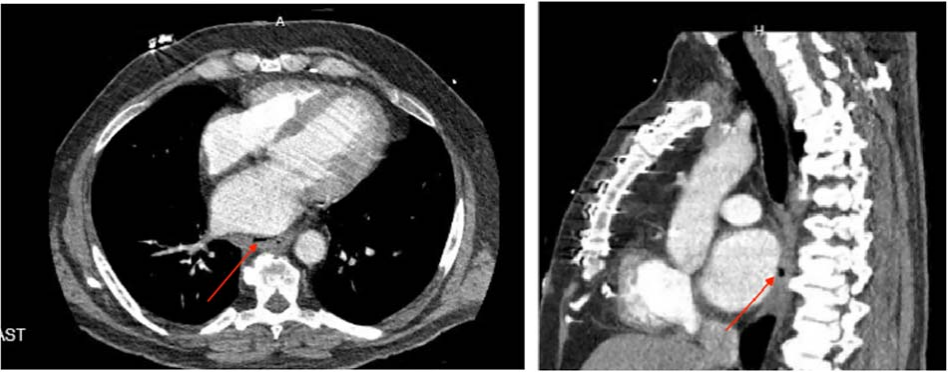
(a) Axial CT image of pericardioesophageal fistula of Case 4 (arrow indicates area of fistulization); (b) sagittal CT image of pericardioesophageal fistula of Case 4 (arrow indicates area of fistulization).

A 58-year-old man, who underwent a left atrial ablation for atrial fibrillation, presented to an outside hospital 4 weeks post-ablation with quadriparesis, chest pain, and fever. A non-contrast CT of the brain revealed infarcts with pneumocephalus. Given his recent ablation, a CT chest was performed, demonstrating a pericardioesophageal fistula (Fig. 2a and b). The patient was started on broad-spectrum antibiotics and was transferred to our institution and taken emergently to the OR, where he underwent a left thoracotomy. Once the pericardium was entered, two small defects in the left atrium were noted, indicating that the atrium was involved in the fistula. These were repaired primarily. Inspection of the esophagus revealed a 3 cm defect, which was also repaired primarily. An intercostal muscle flap was placed between the esophageal repair and the pericardium. During his hospitalization, he had multiple pulseless electrical activity cardiac arrests and a declining neurologic exam. Despite multiple CT scans and esophograms revealing a solid repair with no evidence of a leak, he continued to appear septic, likely due a small, persistent atrioesophageal fistula undetectable by imaging. Following his last arrest, his neurologic status declined significantly, and the decision was made to transition to comfort care, and he subsequently expired.

## Discussion

Catheter ablation was previously used as a last resort for refractory atrial fibrillation, but over the last decade it has increasingly been offered as a therapeutic option to a broader group of patients. The increased application of this treatment is attributed to several factors. Generally, the procedure is well-tolerated and is often performed without complications, especially in high volume centers after several years of development [1, 5, 7]. A recent randomized controlled study demonstrated that catheter-based ablation led to clinically significant improvements in quality of life when compared with pharmacological therapy [8]. A follow-up to this study demonstrated lower recurrence rates of atrial fibrillation compared with medical therapy [9]. With the increasing use of ablation therapy, it is important to be aware of the potential complications. Pericardioesophageal fistulae are rare but can impose significant morbidity on the patient and carry a high mortality rate if not identified and managed early.

Pericardioesophageal fistulae develop due to a thermal injury to the esophagus during the ablation in the left atrium, which is in close proximity to the upper and mid-esophagus. If an injury progresses and goes unrecognized and untreated, it can fistulize to the pericardium and atrium. Patients have variable presenting symptoms, which can make this problem difficult to diagnose. Symptoms such as chest pain, dysphagia, and fever should prompt investigation, but a high index of suspicion is required for timely diagnosis. The timeframe for developing symptoms is variable as well, but patients usually present about 7–35 days post-ablation [2, 10, 11].

In this case series we report on four patients who developed pericardioesophageal fistulae after ablation for atrial fibrillation, including their presentations and outcomes. In order to prevent severe morbidity and even death, early diagnosis and intervention are necessary. The mainstay of treatment is surgical intervention, but there have been cases reported in which esophageal stenting and drainage alone were used treat this complication [12, 13]. However, in an analysis of outcomes, patients with documented perforation and fistula formation, who underwent esophageal stenting instead of surgery, had a significantly higher mortality and development of neurologic injury as compared with those who underwent surgery [1]. In the second case of this series, we utilized esophageal stenting because the perforation was small and had been previously been repaired. In the third case, the perforation was larger with devitalized areas that necessitated debridement, so primary repair was warranted.

When managing these patients, early intervention is important because source control and hemodynamic support are essential to mitigate the clinical deterioration these patients often experience. With regard to the surgical approach, it was our experience that a combined approach of upper endoscopy as well as chest exploration to repair the esophagus and fistula yielded the best outcomes. As a tertiary care center, all cases presented were initially transfers from outside hospitals. However, the delay in recognition of the complication and subsequent transfer for surgical management that some patients received at referring hospitals likely impacted their outcomes. Two of the four patients who survived and ultimately recovered underwent early drainage procedures, in the form of pericardial windows and pleural chest tubes along with broad spectrum IV antibiotics. This provided some source control and likely temporized clinical deterioration until they were transferred to our center for definitive management. This highlights the importance of early diagnosis and attempts at source control. Furthermore, since this complication is complex and requires a multi-specialty approach, sometimes with CPB, it is important that the patients be managed at a tertiary care center with both cardiac and thoracic surgery teams available.

## Conclusion

Ablation for atrial fibrillation is an increasingly common and well-tolerated procedure; however, it is important to be aware of the potential complications that it can cause. Although rare, esophageal injury and fistulization can be devastating and is associated with a high rate of mortality if not recognized and managed early. Temporizing measures, such as drainage and antibiotics, can be life-saving while arrangements are made for the patient to be transferred to a tertiary center for definitive management.
